# Enhancement of resistive switching under confined current path distribution enabled by insertion of atomically thin defective monolayer graphene

**DOI:** 10.1038/srep11279

**Published:** 2015-07-10

**Authors:** Keundong Lee, Inrok Hwang, Sangik Lee, Sungtaek Oh, Dukhyun Lee, Cheol Kyeom Kim, Yoonseung Nam, Sahwan Hong, Chansoo Yoon, Robert B. Morgan, Hakseong Kim, Sunae Seo, David H. Seo, Sangwook Lee, Bae Ho Park

**Affiliations:** 1 Division of Quantum Phases & Devices, Department of Physics, Konkuk University, Seoul, 143-701, Korea; 2 Electronic Materials Research Center, Korea Institute of Science and Technology, Seoul 136-791, Korea; 3 Department of Physics, Sejong University, Seoul, 121-742, Korea; 4 Samsung Advanced Institute of Technology, Samsung Electronics, Yongin, Gyeonggi-do, 466-712, Korea

## Abstract

Resistive random access memory (ReRAM) devices have been extensively investigated resulting in significant enhancement of switching properties. However fluctuations in switching parameters are still critical weak points which cause serious failures during ‘reading’ and ‘writing’ operations of ReRAM devices. It is believed that such fluctuations may be originated by random creation and rupture of conducting filaments inside ReRAM oxides. Here, we introduce defective monolayer graphene between an oxide film and an electrode to induce confined current path distribution inside the oxide film, and thus control the creation and rupture of conducting filaments. The ReRAM device with an atomically thin interlayer of defective monolayer graphene reveals much reduced fluctuations in switching parameters compared to a conventional one. Our results demonstrate that defective monolayer graphene paves the way to reliable ReRAM devices operating under confined current path distribution.

Nowadays one of the most widely used nonvolatile memories is flash memory. It has been employed to numerous mobile devices and becomes a representative product supplied by the Si based semiconductor industries. The flash memory is expected to reach limitations in operating speed, power consumption, and density of memory in near future because it is a charge-storage type memory based on a Si transistor. To overcome the limitations, many researchers have tried to develop next generation nonvolatile memories (NG-NVM) with high performances, which do not rely on stored charges and Si transistors^1^,^2^,^3^,^4^.

ReRAM whose resistive change is induced by applied external electrical stress is considered as one of these NG-NVMs. In addition to the advantageous properties of oxide-based ReRAM such as simple composition, facile fabrication process, and compatibility with conventional semiconductor processes, this resistance-change memory has presented promising nonvolatile memory effects including fast operation speed, low power consumption, and high scalability^5^,^6^,^7^,^8^,^9^.

Resistive switching can be classified into two categories: Uni-polar and bi-polar resistive switching caused by conducting filament formation in bulk and oxygen migration at interface, respectively. Especially, uni-polar resistive switching, which is usually observed in binary oxide, has been explained by conducting filament (CF) mechanism in switching materials. The creation and rupture of CFs presumably result from Joule heating^9^,^10^,^11^,^12^,^13^,^14^, localized phase transition^15^, dislocation^16^,^17^, or defect in grain boundary^18^. However, questions about stability of operation parameters in ReRAM, which are relevant to how to control CFs, have not yet been fully answered. Uniform current path distribution across the switching materials causes randomly created and ruptured CFs leading to large fluctuations of switching parameters during resistive switching operation^19^,^20^,^21^.

In this paper, to reduce fluctuation in switching parameters, we suggest the insertion of a highly defective graphene (d-graphene) monolayer between an oxide film and an electrode. Because localized electronic structure of graphene can be modified by ionic treatments^22^,^23^,^24^,^25^, we control the amount of defects in the graphene monolayer by using an Ar^+^ ion-assisted reaction (IAR) system, which is monitored by Raman spectroscopy^24^,^26^. We have investigated resistive switching under confined current path distribution by d-graphene monolayer between an oxide film and an electrode. Conductive atomic force microscopy (C-AFM) image of d-graphene reveals that the IAR-induced defects in the d-graphene cause confined current path distribution, which may result in suppressed fluctuation of switching parameters in ReRAM devices. The metal/d-graphene/insulator/metal (MGIM) structure provides a good model system which can effectively control the current path distribution in an oxide film and thus achieve its reliable resistive switching.

## Results and Discussion

**Figure 1:**
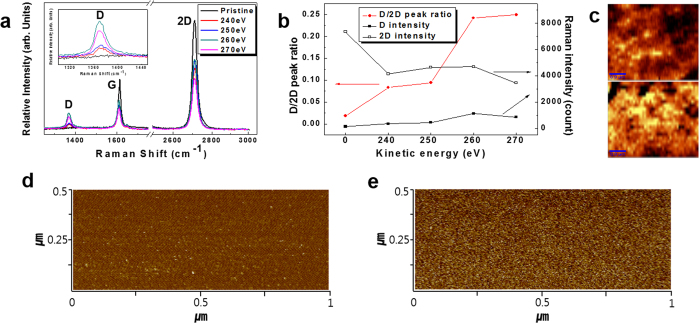
Analysis of d-graphene by Raman spectroscopy and C-AFM. (**a**) Raman spectroscopy of pristine and defective graphenes irradiated with Ar^+^ ions at various kinetic energies. (**b**) D/2D peak ratio and Raman intensity depending on kinetic energy. (**c**) Raman scanning microscopy images of D/G peak ratio obtained from d-graphenes irradiated with Ar^+^ ions at kinetic energies of 240 eV (upper panel) and 270 eV (lower panel). C-AFM images of d-graphenes irradiated with Ar ^+^ ions at kinetic energies of (**d**) 240 eV and (**e**) 270 eV.

Before we fabricate a MGIM structure, we need to confirm quality of our graphene. Raman spectroscopy analysis in Fig. 1a guarantees quality of the as-grown monolayer graphene (MLG) employed in our device, which is fabricated on a Cu/Ni/SiO_2_/Si substrate using chemical vapor deposition (CVD) method (See Materials in Materials and Methods section). In Raman spectroscopy data from honeycomb lattice of graphene, three most considerable features are the G, 2D, and D peaks, which appear around 1580 cm^−1^, 2700 cm^−1^, and 1340 cm^−1^, respectively. Our Raman data only shows G and 2D peaks with G/2D peak ratio of about 0.5, which are very similar with those of intrinsic MLG^24^,^26^. Additionally, scanning electron microscopy image of the graphene on a Cu/Ni substrate with clearly visible grains ensured very high quality of our CVD graphene (Supplementary information, Figure S1)^27^,^28^,^29^,^30^. These observations on pristine MLG clearly support our assumption that most defects on our d-graphene interlayer will be induced by IAR^25^,^30^. The IAR system consists of ion source, sample holder, environmental gas supplier, and pumping system. The Ar^+^ ion beam was generated by a cold hollow cathode-type ion source. Generated number of Ar^+^ ions is measured by Faraday cup placed at a distance of 50 cm from the ion source. Working pressure during Ar^+^ ion bombardment is kept at 0.1 mTorr with Ar flow rate of 5 sccm.

With attempt to provide confined current path distribution, we introduce defects on graphene by bombarding it with Ar^+^ ions at kinetic energies of 240 eV, 250 eV, 260 eV, and 270 eV, respectively, with number of bombarded Ar^+^ ions about 5 × 10^14^ per unit area (cm^2^) and time (second). The 240 eV of applied kinetic energie is minimum value of stably controllable area in our experimental system. Relative defect concentrations in the pristine and defective graphenes can be compared using Raman spectroscopy data, as shown in Fig. 1a. D peaks appear in the Raman spectra obtained from d-graphenes indicating that Ar^+^ ion bombardment induces the breaking of carbon-carbon bonds in graphene honeycomb structure^31^,^32^. Because D peak is associated with disordered carbon atoms or defects and 2D or G peaks are caused by graphene honeycomb structure, D/2D or D/G peak ratios can provide information on the defect concentration^24^,^26^,^33^,^34^. In Fig. 1b, increase of D/2D peak ratio indicates that defect concentration in d-graphene increases with kinetic energy of Ar^+^ ions. To identify the location of defects, we obtained Raman scanning microscopy images of D/G peak ratio in 5 μm × 5 μm scanning areas of two d-graphenes irradiated with Ar^+^ ions at kinetic energies of 240 eV and 270 eV, as shown in the upper and lower panels of Fig. 1c, respectively. Bright spots in the images correspond to defect sites which have potential to generate confined current path distribution^35^. We measured local current distributions on the surface of d-graphenes using conductive atomic force microscope (C-AFM) with a Pt/Ir-coated conductive tip (10 nm radius) under applied bias of 0.1 V. Fig. 1d,e show C-AFM images (1 μm × 0.5 μm) obtained at the surfaces of d-graphenes irradiated with Ar^+^ ions at kinetic energies of 240 eV and 270 eV, respectively. The observed bright spots designate the positions of conducting paths where higher local current (~50 pA) passes through d-graphenes than that (~1 pA) of the other regions. Figure 1e reveals much higher concentration of bright spots than that of Fig. 1d implying that Ar^+^ ion irradiation with lower kinetic energy induces less conducting paths on d-graphene than that with higher kinetic energy. As Jafri *et al.* mentioned, the higher concentration of conducting paths on d-graphene with more defects can be attributed to the defect induced mid-gap states, which create a region exhibiting metallic behavior around the vacancy defects on graphene^35^.

**Figure 2:**
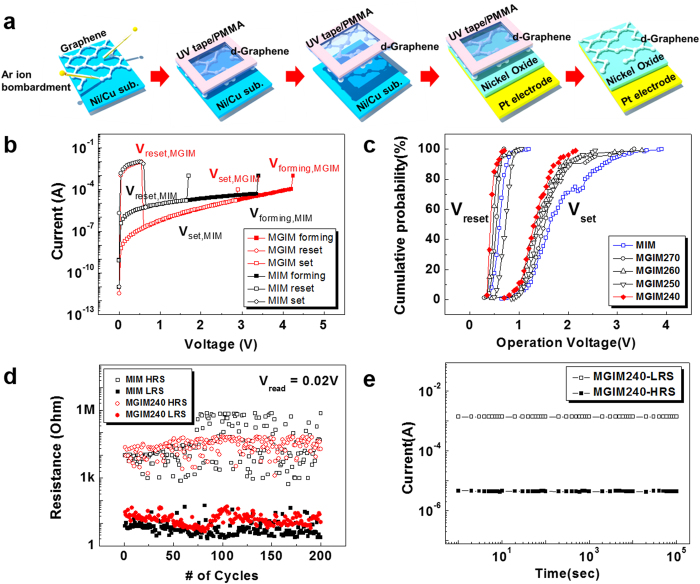
Resistive switching characteristics of MGIM structures compared with a conventional MIM structure. (**a**) Illustration of fabrication process for MGIM structure. D-graphene is made before it is transferred to device. (**b**) Initial current-voltage characteristics of the MGIM and conventional MIM structures. (**c**) Cumulative probability of switching voltages, V_set_ and V_reset_, for MGIM structures with d-graphenes irradiated with Ar^+^ ions at kinetic energies of 240 eV (MGIM240), 250 eV (MGIM250), 260 eV (MGIM260), and 270 eV (MGIM270) as well as a MIM structure. (**d**) Change in resistance states for MGIM240 and MIM, which are measured at room temperature and atmospheric pressure. (**e**) Retention characteristics of MGIM240 measured at 85 °C in a vacuum of 1 mTorr as well as ambient atmospheric condition under reading volate of 0.1 V.

To fabricate a MGIM structure, d-graphene introduced in Fig. 1 is inserted between top Pt electrode and insulating NiO film by using micro contact transfer technique, as illustrated in Fig. 2a. We deposited NiO on Pt/Ti/SiO_2_/Si substrate using dc reactive sputtering method with the same condition of our previous works (See Materials and Transfer of Graphene in Materials and Methods section)^36^,^37^,^38^,^39^,^40^,^41^,^42^,^43^,^44^. After the transfer process, Pt top electrodes with a thickness of 100 nm and an area of 50 μm × 50 μm were fabricated on the GIM structure using dc sputtering method and conventional lift-off process.

Figure 2b shows the typical unipolar resistive switching behaviors of MIM, which is a conventional Pt/NiO/Pt capacitor without d-graphene, and MGIM structures. Each structure stays initially in a high resistance state (HRS). When an applied voltage is swept to the forming voltage (V_forming_), measured current abruptly increases and the structure reaches a low resistance state (LRS). During the following voltage sweep with step voltage of 0.05 V from 0 V, switching from LRS to HRS and switching from HRS to LRS occur at V_reset_ and at V_set_, respectively. Compliance current of 1 mA is considered as a minimum value which can allow forming and reproducible set process in our NiO capacitor systems. The MGIM structure initially shows higher V_set_ value and lower current level of HRS than those of the MIM structure owing to the additional resistance from the inserted d-graphene interlayer. Figure 2c shows the cumulative probabilities of switching voltages for MGIM structures with d-graphenes irradiated with Ar^+^ ions at kinetic energies of 240 eV (MGIM240), 250 eV (MGIM250), 260 eV (MGIM260), and 270 eV (MGIM270) as well as a MIM structure. The cumulative probability for each structure was obtained after over 200 switching cycles. The distributions of the switching voltages, V_set_ and V_reset_, are important parameters indicating memory device performances. According to the cumulative probability data, we can confirm that the switching voltage distributions of MGIM structures are narrower than that of a conventional MIM structure. Especially, MGIM240 shows much narrower distributions of V_set_ (0.5 ~ 2.1 V, standard deviation (SD) of 0.32 V) and V_reset_ (0.3 ~ 0.7 V, SD of 0.08 V) than those of V_set_ (0.6 ~ 4 V, SD of 0.63 V) and V_reset_ (0.4 ~ 1.2 V, SD of 0.12 V) in the MIM structure. Due to such wider distribution, V_set_ of the MIM occasionally becomes higher than that of the MGIM. MGIM240 also shows the narrowest distributions in the cumulative probability plots of resistance values in HRS and LRS states (Supplementary information, Figure S4(a)). The change in resistance states for MGIM240 and MIM during 200 cycles of resistive switching is shown in Fig. 2d. While both devices are successfully operated for 200 cycles of operations, HRS and LRS for MIM fluctuate largely in the ranges of 1 KΩ ~ 1 MΩ (open black square) and 2 Ω ~ 60 Ω (solid black square), respectively, at constant voltage of 0.02 V. The fluctuations of HRS and LRS for MGIM240 are dramatically reduced in the ranges of 1 KΩ ~ 50 KΩ (open red circle) and 5 ~ 50 Ω (solid red circle), respectively. Figure 2e shows the retention characteristics of MGIM240 which ensure that the MGIM structure is able to retain its HRS and LRS states over 10^6^ seconds at 85 °C in vacuum of 1 mTorr under reading voltage of 0.1 V. The retention capability of MGIM240 is not worse than that of a MIM structure, which was reported in a previous report^39^.

**Figure 3:**
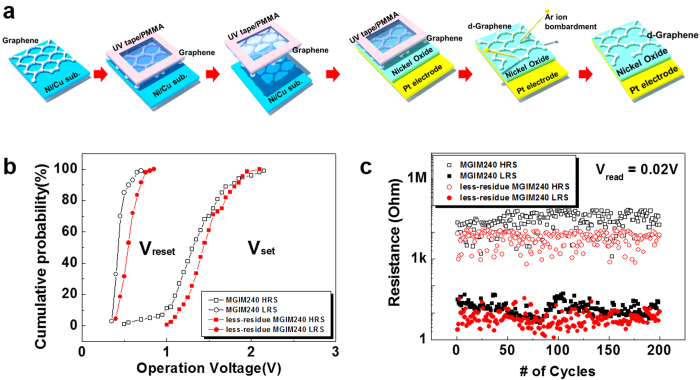
Introduction of less-residue MGIM structures. (**a**) Illustration of modified fabrication process for less-residue MGIM. D-graphene is made after it is transferred to device. (**b**) Cumulative probabilities of switching voltages, V_set_ and V_reset_, and (**c**) change in resistance states for MGIM structures where inserted MLGs are bombarded with Ar^+^ ions at the kinetic energy of 240 eV before (MGIM240) and after (less-residue MGIM240) transfer of the MLGs on NiO films.

In previous reports, graphene devices have shown suppressed performances than those predicted by theory owing to residues, ripples, vacancies, etc^45^,^46^,^47^. To remove the adverse effects of residues caused by conventional transfer process, we modified device fabrication procedure as follows. As shown in Fig. 3a, we performed IAR treatment after transfer of MLG because irradiation with Ar^+^ is able to etch residues as well as induce defects on graphene during IAR treatment. Figure 3b shows cumulative probabilities of switching voltages for MGIM structures where inserted MLGs are bombarded with Ar^+^ ions at the kinetic energy of 240 eV before (MGIM240) and after (less-residue MGIM240) transfer of the MLGs on NiO films. Less-residue MGIM240 shows narrower distribution of V_set_ (1 V ~ 2.1 V, SD of 0.24 V) than that for MGIM240 due to the removal of residues on the MLG during Ar^+^ ion bombardment although distribution of V_reset_ (0.4 V ~ 0.85 V, SD of 0.08 V) for less-residue MGIM240 is comparable to that for MGIM240. Each resistance state for less-residue MGIM240 is stable similarly to that for MGIM240, as shown in Fig. 3c and supplementary information Figures S3 and S4(b).

During the modified fabrication process, Ar^+^ ions bombarded on MLG/NiO may induce defects in the NiO layer as well as on the MLG. According to a previous study about ReRAM treated using IAR^48^, resistive switching characteristics was improved by chemical or structural defects in an oxide layer resulting from Ar^+^ bombardment. It was found that the forming voltage could be controlled by modification of surface roughness and oxygen vacancy concentration of an oxide layer, which depended on kinetic energy of the bombarded Ar^+^ ion. However, in our case, the existence of MLG interlayer might reduce the effect of Ar^+^ bombardment on the underlying NiO layer. To demonstrate the protection role of graphene, we fabricated MIM structures with defective NiO layers directly bombarded by Ar^+^ ions at kinetic energies of 240 eV (DMIM240), 250 eV (DMIM250), and 260 eV (DMIM260).

**Figure 4:**
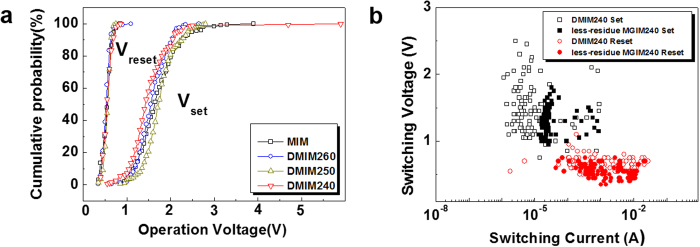
Resistive switching characteristics of DMIM structures without inserted graphene layers. (**a**) Cumulative probabilities of switching voltages, V_set_ and V_reset_ for MIM structures with undamaged (MIM) and irradiated (DMIM240, DMIM250, DMIM260) NiO layers. (**b**) Switching voltage *versus* switching current plots of DMIM240 and less-residue MGIM240 without and with d-graphene layer, respectively.

Figure 4a shows cumulative probabilities of switching voltages for MIM structures with undamaged (MIM) and irradiated (DMIM240, DMIM250, DMIM260) NiO layers. Although switching voltage fluctuations of DMIM series decrease as kinetic energy of Ar^+^ ion increases, they are comparable to those of MIM. In contrast, DMIM240 reveals larger fluctuations in switching voltages and currents than those of less-residue MGIM240 with d-graphene, as shown in Fig. 4b. Therefore, we can argue that the enhanced switching parameter distribution of less-residue MGIM240 is mainly caused by d-graphene layer, instead of defective oxide layer, resulting from Ar^+^ ion bombardment.

When a voltage is applied to the MGIM structure, we assume that the confined current path formed on d-graphene, as shown in Fig. 1d,e, also causes confinement of current path on the surface of NiO layer due to series connection of d-graphene and NiO layer. Through the confined current paths, selective oxygen ion migration may take place^49^, resulting in preferential generation of CFs. It seems that d-graphene irradiated with Ar^+^ ions at kinetic energy of 240 eV has lower conducting path concentration than that of 270 eV leading to more reduced randomness during formation and rupture of CFs in an oxide layer underneath a d-graphene. To vary the number of defects on graphene, we fabricated two MGIM240 structures in which graphenes are bombareded with different numbers of Ar^+^ ions: 3 × 10^14^/cm^2^ and 8 × 10^14^/cm^2^ (Supplementary information, Figure S5). The superior performance of 3 × 10^14^/cm^2^ case than that of 8 × 10^14^/cm^2^ case supports our argument.

## Conclusion

In summary, we propose a new ReRAM device structure (MGIM) simply modified by insertion of a defective graphene layer between a top electrode and on oxide layer. This atomically thin interlayer inserted into an MIM device can reduce randomness during formation and rupture of conducting filaments by confined current path distribution in the modified system. MGIM structures show much narrower range of V_set_ and V_reset_, and more stable resistance states than those of conventional MIM structures. The insertion of atomically thin defective monolayer graphene for the enhancement of ReRAM device performance is a promising method compatible with conventional semiconduting technology because fabrication and transfer of a defective monolayer graphene are easily scaled up to wafer size, and its insertion results in thickness increase by only 0.4 nm.

## Methods

### Materials

Monolayer graphene was synthesized by inductively coupled plasma enhanced chemical vapor deposition (ICP-CVD) on a Cu/Ni/SiO_2_/Si substrate. During the growth process, the substrate is heated to 650 °C within 10 min under ~10^−7^ torr and then treated with H_2_ plasma. After purging with Ar gas for a couple of minutes, C_2_H_2_ is added (C_2_H_2_:Ar = 1:40) for graphene growth at the same temperature. A 35 nm thick polycrystalline NiO thin film was prepared by dc reactive sputtering method on Pt/Ti/SiO_2_/Si substrate (substrate temperature of 500 °C, 1.5 mTorr working pressure of Ar + O_2_ mixture gas, O_2_ ratio of 7%).

### Transfer of Graphene

For the monolayer graphene transfer, graphene/Cu/Ni/SiO_2_/Si was spin-coated with polymethyl methacrylate (PMMA) 950 C4 on which we attached a pressure sensitive adhesive ultraviolet tape. Peeling the tape against the Si wafer physically separated the tape/PMMA/graphene/Cu/Ni layer due to poor adhesion of the metal films to SiO_2_. After etching of the underlying Cu/Ni by soaking in FeCl_3_ and cleaning in water, the tape/PMMA/graphene layer was pressed onto the NiO/Pt/Ti/SiO_2_/Si. The successive removal of the tape and PMMA in ethanol and acetone, respectively, left only the graphene layer on the NiO/Pt/Ti/SiO_2_/Si.

### Characterization

Most electrical properties of the MGIM device (such as resistive switching characteristics and fluctuations of the switching parameters) were measured using a Keithly 2400 sourcemeter at room temperature and atmospheric pressure, but all retention measurements were performed additionally at 85 °C and 1 mTorr. The electrical data were obtained on a single device for each kind of structure, which showed the best performance among several ten devices. The Raman spectroscopy measurements were performed at room temperature with a 532 nm diode-pumped solid state laser and a microscope setup with a laser spot diameter of 200 nm.

## Additional Information

**How to cite this article**: Lee, K. *et al.* Enhancement of resistive switching under confined current path distribution enabled by insertion of atomically thin defective monolayer graphene. *Sci. Rep.* **5**, 11279; doi: 10.1038/srep11279 (2015).

## Supplementary Material

Supplementary Information
