# Supplementary material for: Enhancement of resistive switching under confined current path distribution enabled by insertion of atomically thin defective monolayer graphene
Source: Sci Rep. 2015 Jul 10;5:11279. doi: 10.1038/srep11279 (PMC4498384; doi:10.1038/srep11279)
Supplement: Supplementary Information [file srep11279-s1.doc]

**Supplementary Information**

**Enhancement of resistive switching under confined current path distribution enabled by insertion of atomically thin defective monolayer graphene**

**Keundong Lee^1†^, Inrok Hwang^1,2†^, Sangik Lee^1^, Sungtaek Oh^1^, Dukhyun Lee^1^, Cheol Kyeom Kim^1^, Yoonseung Nam^1^, Sahwan Hong^1^, Chansoo Yoon^1^, Robert B. Morgan^1^, Hakseong Kim^1^, Sunae Seo^3^, David H. Seo^4^, Sangwook Lee^1^ and Bae Ho Park^1*^**

^1^ Division of Quantum Phases & Devices, Department of Physics, Konkuk University, Seoul, 143-701, Korea

^2^ Electronic Materials Research Center, Korea Institute of Science and Technology, Seoul 136-791, Korea

^3^ Department of Physics, Sejong University, Seoul, 121-742, Korea

^4^ Samsung Advanced Institute of Technology, Samsung Electronics, Yongin, Gyeonggi-do, 466-712, Korea

^†^These authors equally contributed to this work.

*Correspondence and requests for materials should be addressed to B. H. P. (email: baehpark@konkuk.ac.kr)

**I. Scanning electron microscope image of monolayer graphene**

Figure S1 shows scanning electron microscope (SEM) image of our monolayer graphene (MLG) deposited on a Cu/Ni/SiO_2_/Si substrate using chemical vapor deposition (CVD) method, where the Cu grains are clearly visible. Some wrinkles of MLG, which may be caused by the thermal expansion coefficient difference between Cu and graphene, are found to cross substrate grain boundaries, indicating continuous graphene film and no other significant macroscopic defects.


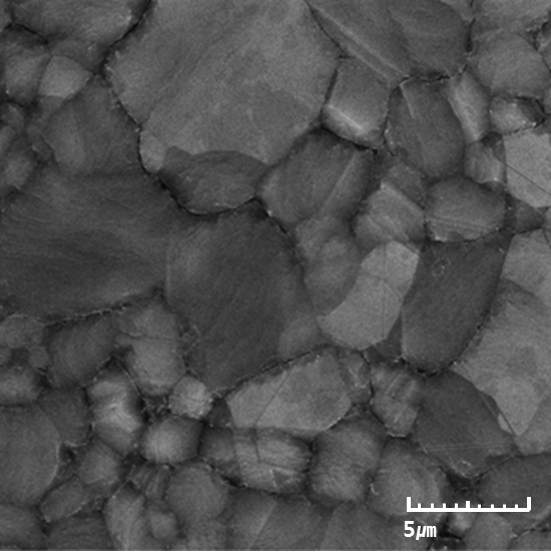


**Figure S1.** **Scanning electron microscope image of monolayer graphene grown on a Cu/Ni substrate.**

**II. The endurance characteristics of MGIM series**

Figure S2 shows the endurance characteristics of Pt/d-graphene/NiO/Pt (MGIM) structures with d-graphene irradiated with Ar^+^ ions at kinetic energies of 240 eV (MGIM240), 250 eV (MGIM250), 260 eV (MGIM260), and 270 eV (MGIM270). All the MGIM structures reveal more stable resistance states than those of a MIM structure (see black squares in Figure 2d). Figure 2 and Figure S2 clearly demonstrate that the d-graphene interlayer causes suppressed switching voltage fluctuation and enhanced resistance stability in MGIM structures.


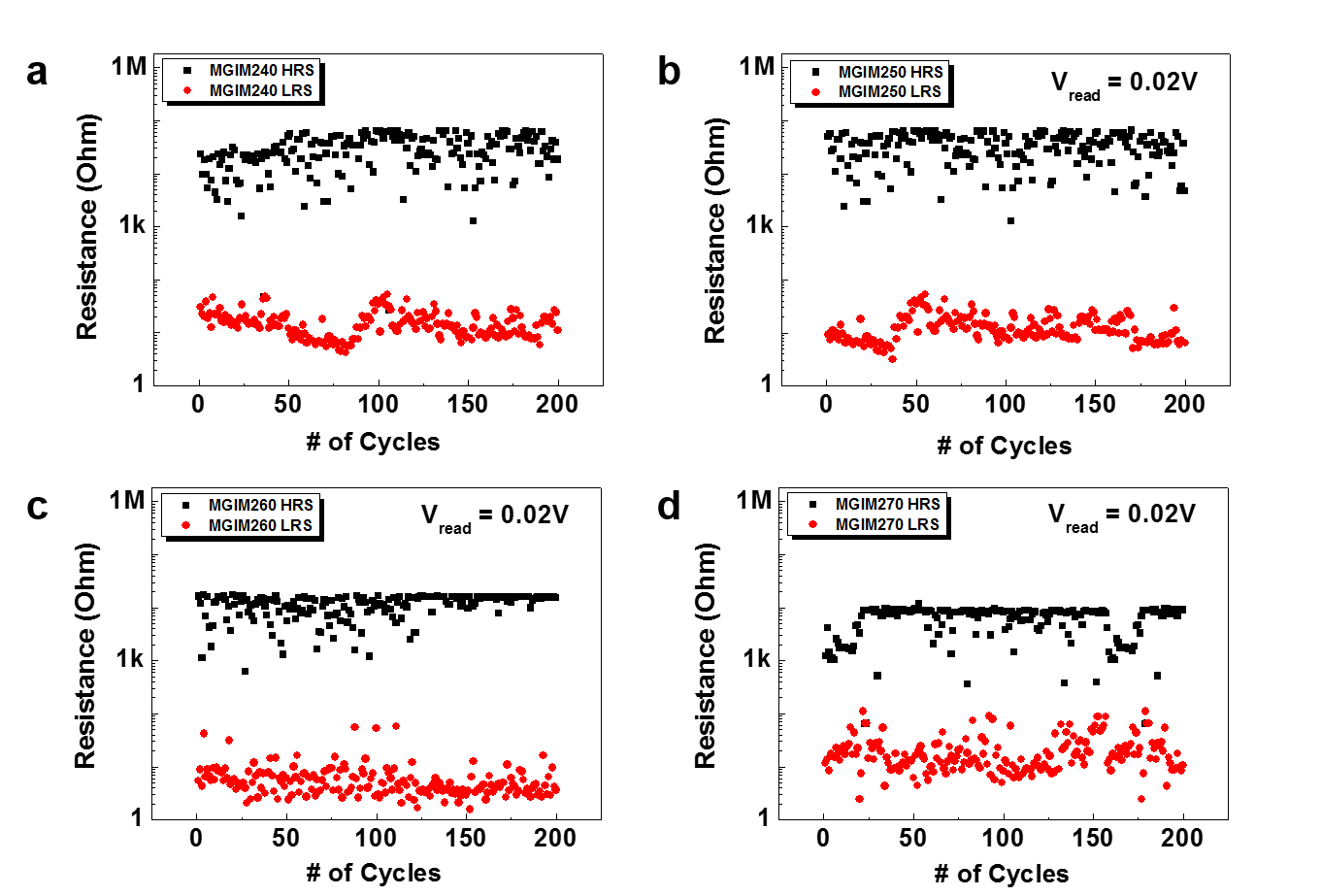


**a**

**b**

**d**

**Figure S2.** **Stability of resistance states for MGIM series.** Endurance characteristics of resistance states for MGIM structures with d-graphene irradiated with Ar^+^ ions at kinetic energies of (**a**) 240 eV (MGIM240), (**b**) 250 eV (MGIM250), (**c**) 260 eV (MGIM260), and (**d**) 270 eV (MGIM270). All of samples show enhanced endurance characteristics over those of conventional MIM structure (see black squares in Figure 2d).

**Ⅲ. Resistive switching characteristics of less-residue MGIM series**

Figure S3(a) shows cumulative probability of switching voltages for less-residue MGIM series with d-graphenes irradiated with Ar^+^ ions at kinetic energies of 240 eV (less-residue MGIM240), 250 eV (less-residue MGIM250), 260 eV (less-residue MGIM260), and 270 eV (less-residue MGIM270) after transfer as well as a MIM structure. Less-residue MGIM series shows decrease in operation voltage fluctuation with kinetic energy of Ar^+^ ions similarly to the MGIM series, as shown in Figure 2(c). Figures S3(b)-(e) exhibit the endurance characteristics of resistance states for less-residue MGIM structures. Each resistance state for less-residue MGIM, whose d-graphene is bombarded with Ar^+^ ions at kinetic energy lower than 260 eV, is stable similarly to that for corresponding MGIM structure. However, less-residue MGIM, whose d-graphene is bombarded with Ar^+^ ions at kinetic energy higher than 260 eV, reveals unstable resistance states probably because Ar+ ions with high kinetic energy may generate damages on not only graphene but also NiO surface underneath it.


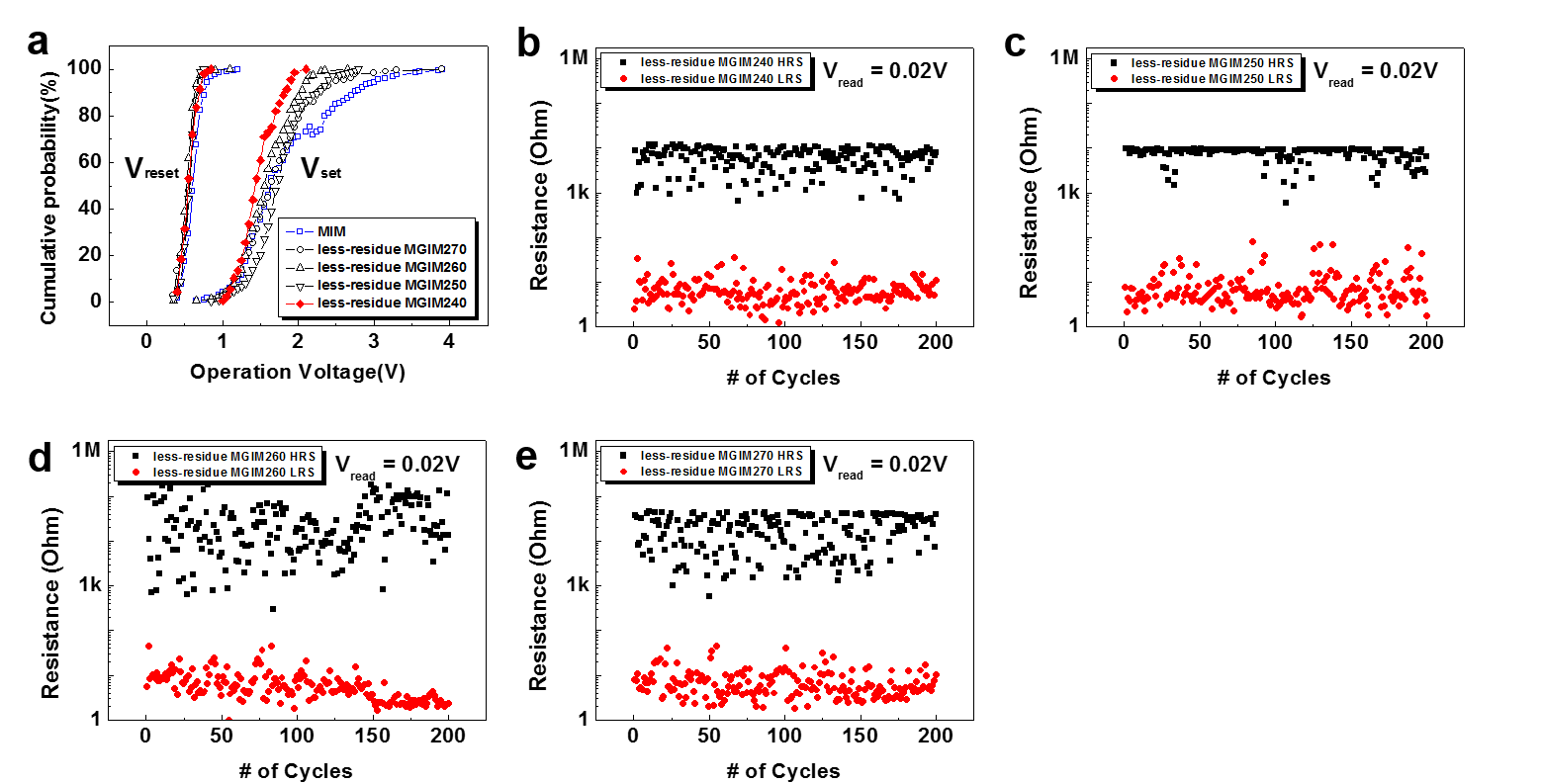


**c**

**(b)**

**(c)**

**(a)**

**Figure S3.** **Resistive switching characteristics of less-residue MGIM series.** (**a**) Cumulative probability of switching voltages for less-residue MGIM structures and a MIM. Resistance stability of less-residue MGIM structures with d-graphenes irradiated with Ar^+^ ions at kinetic energies of (**b**) 240 eV (less-residue MGIM240), (**c**) 250 eV (less-residue MGIM250), (**d**) 260 eV (less-residue MGIM260), and (**e**) 270 eV (less-residue MGIM270) after transfer.

**Ⅳ. Cumulative probability of resistance values**

Figure S4(a) shows cumulative probability of resistance values in HRS and LRS states for MGIM series and a MIM. Large fluctuation in resistance values of the MIM is drastically decreased especially in MGIM240, whose tendency is very similar with that in switching voltages as shown in Figure 2(c). Figure S4(b) illustrates cumulative probability of resistance values for MGIM240 and less-residue MGIM240. The two devices show comparably small fluctuation in resistance values.


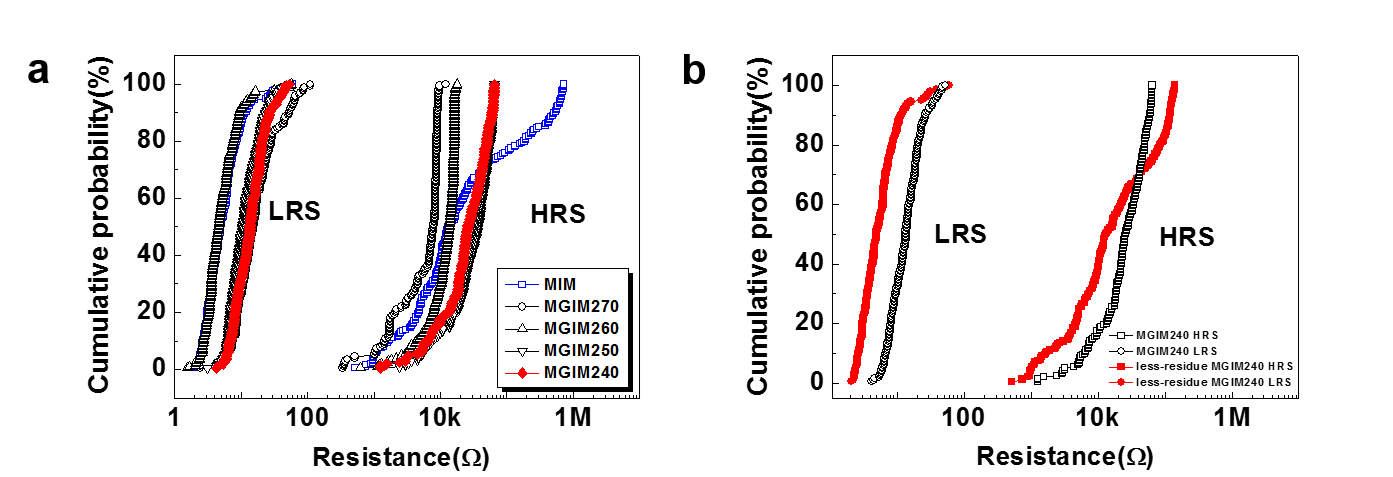


**Figure S4.** **Cumulative probability of resistance values.** Cumulative probabilities of resistance values for (a) MGIM series and a MIM and for (b) MGIM240 and less-residue MGIM240.

**Ⅴ. Variation of number of irradiated Ar^+^ ions**

To confirm the dependence of device performance on the number of defects in graphene, d-graphene is created by different number of irradiated Ar^+^ ions using control of irradiation time at constant kinetic energy of 240 eV. Figure S5(a) shows Raman spectroscopy of d-graphene irradiated with 3 × 10^14^/cm^2^ and 8 × 10^14^/cm^2^ Ar^+^ ions. D-graphene irradiated with 3 × 10^14^/cm^2^ ions shows smaller D/2D peak ratio than that with 8 × 10^14^/cm^2^ ions implying lower number of defects. Figure S5(b) exhibits cumulative probability of switching voltages of two MGIM240 devices in which d-graphenes are irradiated with 3 × 10^14^/cm^2^ and 8 × 10^14^/cm^2^ Ar^+^ ions. The MGIM240 device in which d-graphene is irradiated with 3 × 10^14^/cm^2^ shows narrower distribution of switching voltages than those with 8 × 10^14^/cm^2^ supporting the argument that lower number of defects on d-graphene leads to more reduced randomness during formation and rupture of CFs in an oxide layer underneath a d-graphene.


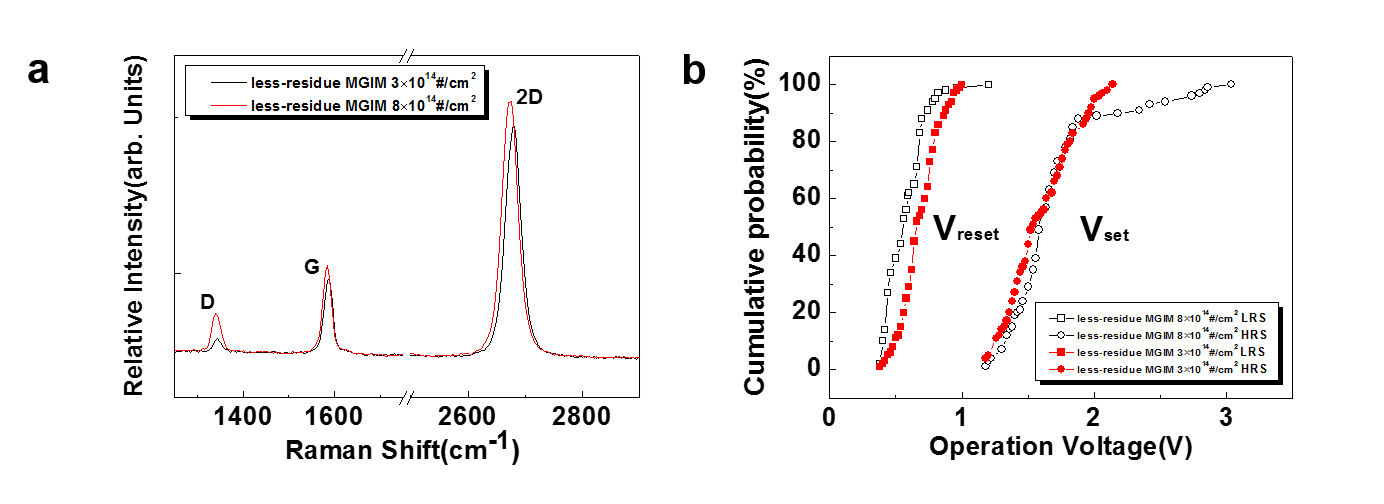


**Figure S5.** **Variation of number of irradiated Ar^+^ ions.** (a) Raman spectroscopy of d-graphenes and (b) cumulative probability of switching voltages of MGIM240 devices in which d-graphenes are irradiated with 3 × 10^14^/cm^2^ and 8 × 10^14^/cm^2^ Ar^+^ ions at kinetic energy of 240 eV.

**Ⅵ. Device to device variability**

Figure S6 shows cumulative probability of switching voltages for five sets of less-residue MGIM structures with d-graphenes irradiated with Ar^+^ ions at kinetic energies ranging from 240 eV to 270 eV. The five sets of devices show similar tendencies with those illustrated in Figure S3(a). As kinetic energy of the bombarded Ar^+^ ion is decreased, the ranges of operation voltages are also reduced.

**
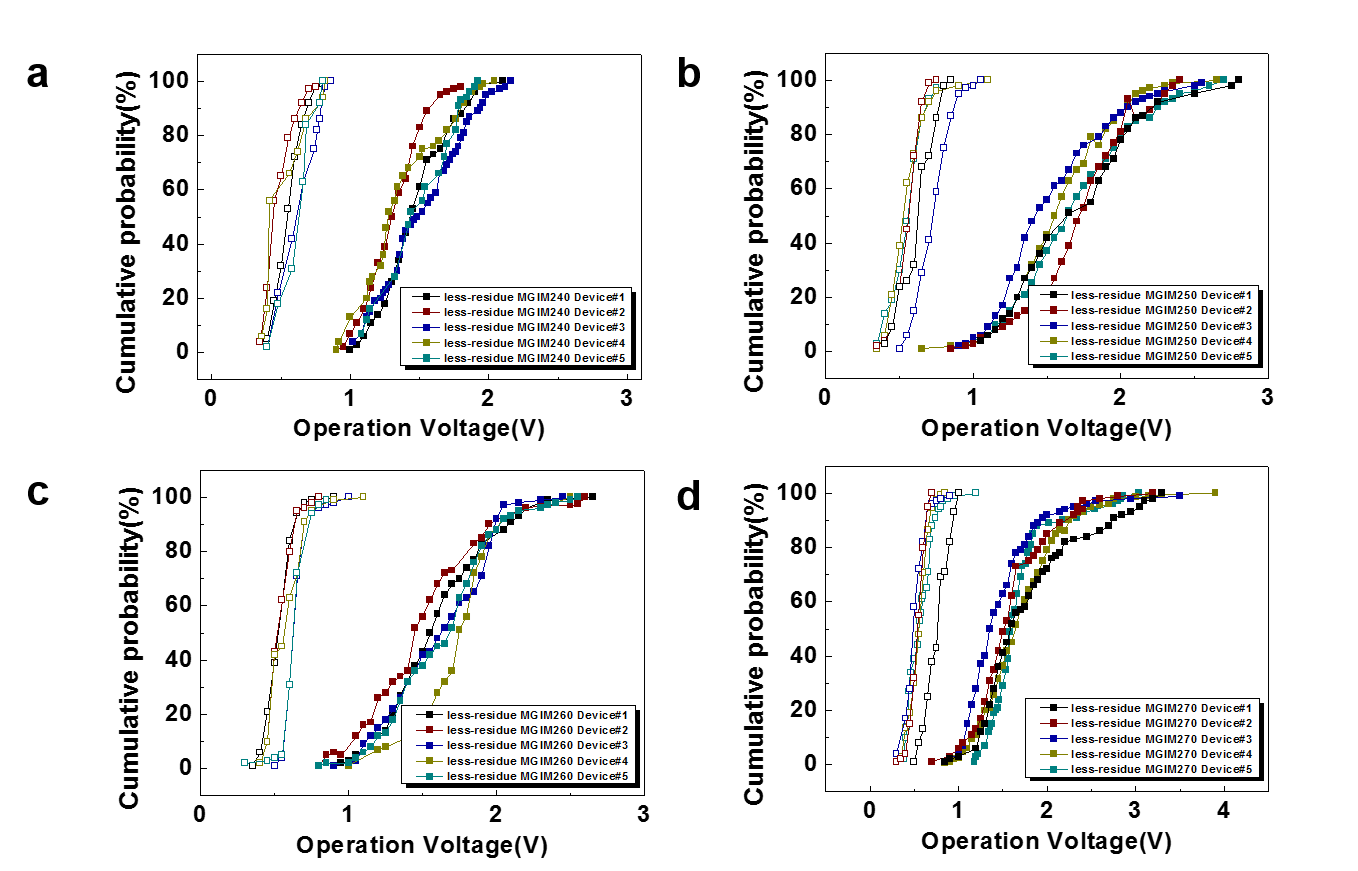
 Figure S6.** **Device to device variablity.** Cumulative probability of switching voltages for five sets of less-residue MGIM structures with d-graphenes irradiated with Ar^+^ ions at kinetic energies of (**a**) 240 eV (less-residue MGIM240), (**b**) 250 eV (less-residue MGIM250), (**c**) 260 eV (less-residue MGIM260), and (**d**) 270 eV (less-residue MGIM270).
